# Essential Oil Blend and Ascorbic Acid Supplementation Improves Performance, Semen Characteristics, Redox Balance, and Intestinal Integrity in Heat-Stressed Male Rabbits

**DOI:** 10.3390/vetsci13050453

**Published:** 2026-05-06

**Authors:** Haifa Ali Alqhtani, Huda A. Alqahtani, Ahmed M. Elbaz, Ahmed Ateya, AbdelRahman Y. Abdelhady, Fatmah Ahmed Safhi, Mohammed Al-Rasheed, Mahmoud H. Mohamed, Wael M. El-Deeb, Mohamed Abdo Rizk, Zakriya Al Mohamad, Mohamed Marzok

**Affiliations:** 1 Department of Biology, College of Science, Princess Nourah bint Abdulrahman University, P.O. Box 84428, Riyadh 11671, Saudi Arabia; haalqhtani@pnu.edu.sa (H.A.A.); faalsafhi@pnu.edu.sa (F.A.S.); 2 Department of Zoology, College of Science, King Saud University, P.O. Box 2455, Riyadh 11451, Saudi Arabia; hudalqahtani@ksu.edu.sa; 3 Animal and Poultry Nutrition Department, Desert Research Center, Cairo 11753, Egypt; dm.a.baz@gmail.com; 4 Department of Development of Animal Wealth, Faculty of Veterinary Medicine, Mansoura University, Mansoura 35516, Egypt; 5 Poultry Production Department, Faculty of Agriculture, Ain Shams University, Cairo 11759, Egypt; abdelrahman_abdelhady@agr.asu.edu.eg; 6 Department of Clinical Sciences, College of Veterinary Medicine, King Faisal University, P.O. Box 400, Al-Ahsa 31982, Saudi Arabia; malraheed@kfu.edu.sa (M.A.-R.); mhmohammad@kfu.edu.sa (M.H.M.); weldeeb@kfu.edu.sa (W.M.E.-D.); mrizk@kfu.edu.sa (M.A.R.); zalmohammad@kfu.edu.sa (Z.A.M.)

**Keywords:** essential oil, ascorbic acid, rabbits performance, gene expression, immunity, semen quality, gut health, heat stress

## Abstract

This study aimed to investigate the effects of supplementing with an essential oil blend and ascorbic acid on performance, semen characteristics, antioxidant status, gut microbiota, immunity, and gene expression in heat-stressed male rabbits. One hundred and forty male New Zealand White rabbits, aged 6 months, were randomly distributed into four dietary groups: the control group receiving basal feed (CON), the group receiving an essential oil blend (EOB, 200 mg/kg), the group receiving ascorbic acid (ASA, 1000 mg/kg), and the group receiving an essential oil blend and ascorbic acid (MAO). The experimental period lasted for eight weeks. The MAO mixture supported rabbits’ tolerance to heat stress by enhancing stress markers, as demonstrated by decreased glucose and HSP70 and increased triiodothyronine (T3). In addition, increased body weight, carcass weight, and nutrient digestibility, but reduced mortality rate, were observed in rabbits fed the MAO mixture. Additionally, semen density and volume, as well as sperm progressive motility and normality, were enhanced in rabbits fed the MAO mixture. Furthermore, MAO mixture supplementation decreases plasma cholesterol, triglycerides, AST, urea, and creatinine levels, while raising HDL and total protein levels. Adding MAO mixture contributed to an increase in plasma SOD and GPx levels, as well as seminal fluid TAC and GSH levels. Additionally, the incorporation of MAO increases IgA, IgG, and IL-10 levels while decreasing IL-6 and TNF-α levels. Adding the MAO mixture reduced *C. perfringens* and *E. coli*, as well as increased the expression of the MUC-2, CAT-1, and CLDN-1 genes. Combining an essential oil blend and ascorbic acid may contribute to improvements in performance, semen quality, immune response, antioxidant capacity, and gut health in heat-stressed male rabbits.

## Simple Summary

Despite significant advances in rabbit production techniques aimed at mitigating heat stress, it remains a major environmental challenge for rabbit breeders. Exposure to elevated temperatures disrupts physiological homeostasis and induces oxidative stress, which may negatively affect growth performance, immune function, gut health, and reproductive efficiency. Consequently, dietary strategies have gained increasing attention as potential approaches to alleviating the adverse effects of heat stress. In this context, the present study investigated the potential benefits of dietary supplementation with a combination of essential oils and ascorbic acid in heat-stressed male rabbits. Specifically, we evaluated whether this nutritional strategy could attenuate oxidative damage and influence productive and physiological responses. The effects of supplementation on growth performance, nutrient digestibility, gut microbiota composition, immune response, antioxidant status, and semen quality were assessed. The findings suggest that the combined antioxidant and immunomodulatory properties of the essential oil blend and ascorbic acid may contribute to improvements in health status, physiological stability, and reproductive performance in heat-stressed male rabbits.

## 1. Introduction

Rabbit production is an important source of food security, sustainable agricultural practices, and income generation, particularly in developing and resource-limited regions [1]. According to data from the Food and Agriculture Organization (FAO), global rabbit meat production has increased steadily over the past few decades. Moreover, rabbits are distinguished by the quality of their meat, rapid growth, efficient feed utilization, early maturity, and high reproduction rate [2]. In addition, rabbits provide a valuable source of high-quality animal protein, which is characterized by low levels of fat and cholesterol and a composition rich in fatty acids, making it an attractive option for health-conscious consumers [3]. Moreover, in developing countries, rabbit farming plays a crucial role in improving rural livelihoods because of its relatively low capital investment, small footprint, ability to be efficiently integrated into small and domestic production systems, and adaptability to various environmental conditions [4].

Rabbits exhibit optimal physiological performance at a moderate temperature range of 18–21 °C, which minimal energy is consumed for thermoregulation, supporting optimal production [5]. The normal body temperature of rabbits is known to typically ranges between 37.7 and 39.0 °C [6,7], although this may vary depending on environmental conditions, breed differences, and measurement methods [8]. Additionally, intensive economic production of rabbits is affected by many factors, including environmental, nutritional, and disease-related factors [9]. During the summer, heat stress leads to a range of physiological and immunological changes in rabbits’ body defense responses, negatively impacting growth performance, reproductive characteristics, and meat quality [10], especially in males. Furthermore, genetically enhanced male rabbits exhibit rapid growth and higher metabolic rates, fewer sweat glands, and denser fur, making them more sensitive to stress [9]. Heat-stressed rabbits alter their physiological responses, exhibiting a significant decrease in thyroid hormone levels in the hypothalamus [8] and thyroid-stimulating hormone levels. Additionally, glucocorticoid synthesis and secretion increase through increased adrenocorticotropic hormone (ACTH) secretion [11]. These changes in the hypothalamic–pituitary–adrenal (HPA) axis result in a marked decrease in metabolic rate, heat production, and sperm quality [9]. One of the most significant negative effects of heat stress is oxidative stress, in which lipid peroxidation increases due to increased free radical activity. This negatively impacts sperm quality, motility, fertility, and function. From the above, it is clear that heat stress negatively affects the reproductive performance of male rabbits, leading to testicular atrophy, increased production of dead and deformed sperm, and a decrease in sperm count [12], thus deteriorating sperm characteristics. In this context, interest has increased in feed additives as an efficient means to reduce the damage caused by heat stress [13,14,15], including the use of probiotics, vitamins, and natural antioxidants [16].

Ascorbic acid (vitamin C) has been proven effective as a feed additive for preventing heat stress, owing to its ability to scavenge free radicals and support oxidative stability [17]. It possesses anti-stress and antioxidant properties [18] via participation in redox reactions. Vitamin C also protects tissues from oxidative damage by eliminating free radicals generated by cell peroxidation during heat stress [17,19]. Additionally, numerous reports indicate that adding ascorbic acid to water or feed enhances growth performance, immunity, and antioxidant capacity [17].

Medicinal and aromatic plants possess numerous properties that support human and animal health and performance [20,21]. Interest in using aromatic plants and their products in poultry feed has increased owing to their diverse natural components, which have proven immune-modulating, antioxidant, antimicrobial, anti-inflammatory, and growth-promoting effects [22,23]. Among their advantages is their effective anti-heat stress effect and potential as an alternative to antibiotics [24]. In rabbit production, these plant additives have been extensively studied for their ability to improve growth performance, enhance immune responses, and support gut health under normal conditions [25]; however, there are limited studies on their effects during heat stress. Among the essential oils that previous reports have shown to play an effective role in alleviating the harmful influences of heat stress are lemon and garlic oils [26], by reducing pathogens and free radicals, in addition to their effect on modulating gene expression [27]. Lemon essential oil primarily consists of limonene, along with beta-pinene and gamma-terpene [28], whereas garlic essential oil is rich in organosulfur compounds, particularly allicin and its derivatives [29], which are responsible for its biological properties. Therefore, the use of medicinal and aromatic plants represents a promising nutritional strategy to promote rabbit health and productivity in sustainable production systems.

Based on previous reports on the positive effects of essential oils and ascorbic acid, we hypothesized that adding them as dietary supplements would enhance performance, feed utilization, antioxidant capacity, immune function, and sperm characteristics in male rabbits exposed to heat stress. However, information available on the combined effects of these additives under conditions of thermal stress is limited. Therefore, this study was designed to address this gap by providing new insights into their synergistic effects and mechanisms of action. The present study aimed to evaluate the impact of adding an essential oil blend, ascorbic acid, and their mixture on performance, sperm characteristics, immunity, redox status, and gut health of male rabbits during heat stress.

## 2. Materials and Methods

### 2.1. Diets, Rabbits, and Management

This study was conducted at the Desert Research Center in Egypt. All instructions for maintaining the safety and welfare of the experimental animals (protocol no. 5-2026-54) were approved by the Faculty of Agriculture, Ain Shams University. Accordingly, the health and well-being of the rabbits were monitored throughout the experimental period. The experiment was conducted from May to June 2025 at the Ras Sadr Research Station, South Sinai Governorate, affiliated with the Desert Research Center, Egypt (https://maps.app.goo.gl/GemxYXTbLRYaJRKbA, accessed on 15 January 2025). One hundred and forty male New Zealand White rabbits, aged 6 months, were placed in individual cages (40 × 60 × 45 cm) and randomly distributed into experimental groups for a 2-week acclimatization period. All animals were in good health at the start of the experiment and had not received any treatments, antibiotics, or growth-stimulating supplements for at least two weeks prior to the start of the experiment. They were provided with controlled environmental conditions and a standard lighting regimen to achieve a stable physiological state. Each group consisted of 35 male rabbits (average initial body weight of 2951 ± 11.3 g) in seven replicates. During the 8-week experiment, unlimited quantities of pellet feed and automatic water were provided daily. The experimental groups were the control group (CON), which was fed a standard diet; the ascorbic acid group (ASA), which was fed a standard diet supplemented with 1000 mg/kg of ascorbic acid; the essential oil blend group (EOB), which was fed a standard diet supplemented with 200 mg/kg of essential oil blend (lemon and garlic); and the mixture group (MAO), which was fed a standard diet supplemented with a mixture of ascorbic acid and essential oils (1000 mg/kg and 200 mg/kg, respectively). The experimental rabbits had free access to a pellet-forming diet and fresh drinking water, and the rabbits were fed a diet designed to meet the nutritional requirements of the National Research Council [30], as shown in Table 1. The welfare and health of the rabbits were carefully monitored daily. Observations included feed and water intake, general behavior, posture, grooming, and any signs of illness or stress. Environmental parameters, such as ambient temperature and humidity, were continuously monitored. In addition, any rabbits showing signs of disease were promptly evaluated and managed according to standard veterinary care procedures, ensuring ethical treatment throughout the study. The rabbits were placed in a well-ventilated facility, where ambient temperature and relative humidity were recorded twice daily (morning and evening) to ensure the welfare of the rabbits. the ambient temperature was 28 ± 3.7 °C and the relative humidity was 56 ± 2.4%. Air circulation was ensured through natural ventilation supplemented by ceiling fans, which maintained uniform airflow throughout the facility. Additionally, for heat-stress evaluation, measurements recorded during the peak midday period (1:00–2:00 p.m.) were used to determine the extent of exposure, as this period represents the highest environmental stress for the animals to calculate the temperature-humidity index (THI), as described by Elbaz et al. [31], to determine the extent of rabbit stress exposure. In addition, the experimental animals were monitored daily to track their overall health, behavior, feeding activity, and signs of stress. No abnormal behavioral responses or signs of aversion to the supplements were observed, and the rabbits remained in good health throughout the experiment, although some symptoms of stress appeared during the afternoon due to the high temperatures. Artificial fluorescent lamps provided lighting with a light/dark ratio of 16L:8D and an intensity of approximately 150 lux at the cage level. The experimental unit was defined according to the measured variable. For growth performance parameters, the replicate group (*n* = 7 per treatment) was considered the experimental unit. For individual-based measurements, including blood biochemical parameters, carcass traits, microbial counts, and gene expression analysis, the individual rabbit was considered the experimental unit.

### 2.2. Preparing Experimental Supplements

L-Ascorbic acid (99%) was obtained from Nanjing Jiayi Sunway Chemical Co., Ltd. (Sunway), Jiangning District, Nanjing City, Jiangsu Province, China. The lemon and garlic essential oils were purchased from CAP PHARM, Cairo, Egypt, a company specializing in the extraction of natural and cosmetic oils. Essential oils were used in their pure form, without the addition of a carrier, and were mixed at a 1:1 ratio (lemon: garlic) before incorporation into the diet at a rate of 200 mg/kg, as described in a previous study [26]. However, no independent analysis (such as GC-MS) was performed to standardize the batch; rather, the chemical composition provided by the manufacturer was adopted. The essential oils were commercial products derived from lemon (*Citrus limon* L., Rutaceae) and garlic (*Allium sativum* L., Amaryllaceae). According to the manufacturer’s specifications, lemon essential oil is extracted from lemon peel through cold pressing. It is characterized by its high content of monoterpenes, particularly limonene (65–75%), as well as minor components such as β-pinene (10.7%) and γ-terpinene (9.3%), α-Pinene (2.1%), and myrcene (1.6%), along with trace amounts of other volatile compounds. Garlic essential oil, on the other hand, is extracted by steam distillation from garlic cloves and is rich in organosulfur compounds, including diallyl disulfide (47.5%), diallyl trisulfide (31.7%), allyl methyl trisulfide (8.6%), diallyl sulfide (4.3%), allyl methyl disulfide (2.9%), and with trace amounts of other volatile compounds. These extraction methods are commonly used to preserve the bioactive constituents and ensure the stability of volatile compounds. Essential oils and ascorbic acid were added directly to the feed by mixing them with a small portion of the base feed, which was then blended into the entire feed using a horizontal mixer under uniform mixing conditions. However, no carriers were used when mixing the essential oils to avoid the potential confounding effects associated with carriers independently, which could affect nutrient absorption or intestinal function. Additionally, the animals were carefully monitored throughout the experiment to observe their feeding behavior and overall feeding activity, ensuring no feed selection or refusal.

### 2.3. Performance Index

Respiratory rate (breaths/min, RR) was recorded daily in the morning by counting the number of breaths per minute. Rectal temperature (RT) of ten rabbits from each group was measured daily by inserting a clinical digital thermometer into the rectum. The rabbits were weighed before the start of the experiment (initial body weight, IBW) and weekly before feeding (final body weight, FBW) each morning to assess the effects of dietary treatments, stress, and feed intake. Weekly feed intake (FI) was calculated by subtracting uneaten feed from the total amount of feed provided each week, and the average daily feed intake (g/day) was calculated during the experimental period. Five rabbits from each group (a total of 20 rabbits) were slaughtered for carcass characteristics and blood measurements. Slaughter was performed by cervical dislocation followed by exsanguination, in accordance with established guidelines for the humane euthanasia of rabbits. All procedures were conducted under the supervision of a veterinarian to ensure minimal stress and pain, in compliance with institutional and international animal welfare regulations. Carcass weight, liver, kidney, lungs, heart, and abdominal fat were measured. Five rabbits were randomly removed from each group at the end of the experiment and placed in individual digestive cages. The rabbits were kept for 24 h to acclimatize before the 5-day fecal collection period, which involved collecting feces three times daily. Feed intake was calculated during the experimental period. The feces were dried, ground, and stored at −10 °C in polyethylene bags. Dry matter, crude fiber, crude protein, ether extract, and nitrogen-free extract were evaluated based on AOAC [32] analysis of the feces and feed. As indicated by Abdel Moneim et al. [15], the digestive enzyme activity (cellulase, trypsin, and amylase) was measured in the intestinal samples collected during fecal collection using commercially available kits (Nanjing Jiancheng Institute of Bioengineering).

### 2.4. Blood Chemistry Indicators

At the end of the experimental period, five male rabbits were slaughtered to collect blood samples for the estimation of the lipid profile, immune response, and oxidative status. After the blood samples were drawn, the plasma was centrifuged and subsequently sent to the laboratory for necessary analyses. Antioxidant enzyme activity was estimated using specialized commercial kits (Bio-diagnostics, Giza, Egypt), including superoxide dismutase (SOD), glutathione peroxidase (GPx), and malondialdehyde (MDA). In addition, immune and inflammatory responses were detected by measuring immunoglobulin levels (IgG, IgM, and IgA) and inflammation-related cytokines, including tumor necrosis factor-alpha (TNF-α), interleukin 6 (IL-6), and interleukin 10 (IL-10), as described by El-Ratel et al. [18]. ELISA kits for inflammatory biomarkers were obtained from MyBioSource (San Diego, CA, USA). Cholesterol, high-density lipoprotein (HDL), low-density lipoprotein (LDL), triglycerides, glucose, total protein, albumin, creatinine, urea, aspartate aminotransferase (AST), and alanine aminotransferase (ALT) concentrations were assayed as biochemical markers of liver and kidney function, as indicated by Elbaz et al. [31]. Moreover, as described by Ibrahim et al. [33], triiodothyronine (T3) was assayed as a performance marker of thyroid function using radioimmunoassay (RIA) kits from Siemens Healthcare (Munich, Germany), and the analysis was performed in accordance with the manufacturer’s instructions.

### 2.5. Semen Collection and Evaluation

During the experimental period, semen was collected from ten rabbits from each group twice a week (at 9:00 a.m.), on Sundays and Wednesdays, throughout the duration of the experiment, to evaluate semen characteristics. Semen was collected repeatedly from the same animals throughout the experimental period. To avoid pseudo-replication, repeated measurements for each animal were averaged before statistical analysis. In addition, ejaculates from two rabbits were pooled to obtain sufficient sample volume for laboratory analyses; thus, each pooled semen sample represented one experimental unit for semen quality evaluation. The collection process was maintained at the same location and time to ensure sample homogeneity. After the collection period, male rabbits were trained to collect semen using an artificial vagina. Immediately after collection, the volume of each ejaculate (after removing the gelatinous mass) was recorded using a graduated collection tube, and sperm density, progressive motility, and viability were assessed, as described by Huang et al. [12]. Additionally, the pH of the semen was determined in 5 mL using pH paper (Merck, Darmstadt, Germany). The semen was transported to the laboratory after being stored in a water bath (37 °C), taking care to protect it from direct light and any thermal shock. Using phase-contrast microscopy, the percentages of viability, normality, and abnormalities were determined after staining the samples with eosin (5%), as indicated by Imbabi et al. [34].

### 2.6. Seminal Plasma

After semen collection, a portion was centrifuged at 700× *g* for 20 min to obtain seminal plasma. This plasma was then immediately sent (to a tank at −20 °C) for oxidative stress analysis. Using a commercial kit (Bio-diagnostics, Giza, Egypt), malondialdehyde (MDA) content, reduced glutathione (GSH), and total antioxidant capacity (TAC) were determined, as per the manufacturer’s instructions, as indicated by Hosny et al. [35]. Additionally, using a commercial ELISA kit (Enzyme-linked, Shanghai, China), heat shock protein 70 (HSP70) was measured, as described by Imbabi et al. [34], following the manufacturer’s recommendations.

### 2.7. Cecum Microbial Count

Five grams of Cecal contents were collected from 20 experimental rabbits (5 rabbits from each group) during slaughter, placed in sterile bags, and stored at −20 °C until microbial count analysis. At the start of the analysis, the necessary 10% (*w*/*v*) dilutions were prepared in sterile peptone solution for 1 h, then sequentially diluted and cultured on appropriate agar for each target microorganism under standard culture conditions, as described by Abdel-Moneim et al. [36] and Elbaz et al. [37] (Table 2). The number of target microbes was expressed as log10 colony-forming units per gram of cecal contents.

### 2.8. Genes Analysis

According to the manufacturer’s instructions, Total RNA was extracted from frozen cecum tissues from five rabbits per group using the QIAamp RNeasy Mini Kit (Qiagen, Hilden, Germany). Approximately 30 mg of tissue was homogenized in lysis buffer and subjected to RNA purification using silica membrane spin columns. RNA concentration and purity were assessed using a NanoDrop 2000 spectrophotometer (Thermo Scientific Inc., Waltham, MA, USA), and samples with A260/A280 ratios between 1.8 and 2.0 were used for downstream analysis. Complementary DNA (cDNA) was synthesized, and quantitative real-time PCR (qRT-PCR) was performed using the QuantiTect SYBR Green RT-PCR Kit (Qiagen, Hilden, Germany). Each reaction (20 µL total volume) contained a SYBR Green master mix, gene-specific forward and reverse primers (0.5 µM each), RNA template, reverse transcriptase enzyme, and nuclease-free water. Amplification was conducted under the following cycling conditions: reverse transcription at 50 °C for 30 min, initial denaturation at 95 °C for 15 min, followed by 40 cycles of denaturation at 94 °C for 15 s, annealing at 55–60 °C for 30 s, and extension at 72 °C for 30 s. The mRNA expression levels of genes encoding nutrient transporters and tight junction proteins, including cationic amino acid transporter-1 (CAT-1), claudin-1 (CLDN-1), and mucin-2 (MUC-2), as well as the pro-inflammatory cytokine interleukin-1β (IL-1β), and interferon-gamma (IFN-γ) levels were evaluated. The specificity of amplification and the absence of primer-dimer formation were confirmed by post-amplification melting curve analysis. Primer sequences for all investigated genes are presented in Table 3. The forward (5′ → 3′) and reverse (3′ → 5′) primers were designed based on published rabbit gene sequences obtained from the NCBI GenBank database and synthesized commercially (Qiagen, Germany). Primer specificity was verified using BLAST analysis 2.17.0 to ensure gene-specific amplification. Using GAPDH (housekeeping gene glyceraldehyde-3-phosphate dehydrogenase) as an internal control, the relative expression levels of the target genes were calibrated. The 2^−ΔΔCt^ technique was used to calculate relative gene expression levels [41].

### 2.9. Statistical Analysis

The general linear model (GLM) algorithm in the SPSS software (version 19.0; SPSS Inc., Chicago, IL, USA) was used to evaluate the data using one-way analysis of variance (ANOVA) in accordance with a randomized complete design. Experimental units varied by measurement: replicates for growth performance parameters, individual rabbits for other traits, while pooled semen samples (two rabbits per sample) for semen analysis, with repeated measures averaged per animal before analysis. Multiple comparisons were conducted using Tukey’s post hoc test to evaluate the difference between CON and other experimental groups under heat stress. Synergistic effects were assessed by comparing the combined treatment mean with the additive effect of individual treatments (mean comparisons) within a one-way ANOVA, with differences interpreted based on the direction and magnitude of deviation relative to group variability (standard error). Normality and homogeneity of variance were assessed using the Shapiro–Wilk test. Differences were considered statistically significant at *p* < 0.05. Data are presented as mean ± SD. Data were analyzed using the following general linear model: Yij = μ + Ti + eij.

Where Yij; observed value of the dependent variable, μ; overall mean, Ti; fixed effect of the ith dietary treatment (i = 1–4), eij; random error associated with each observation, assumed to be independent and normally distributed.

## 3. Results

### 3.1. Thermoregulatory Response

**Figure 1:**
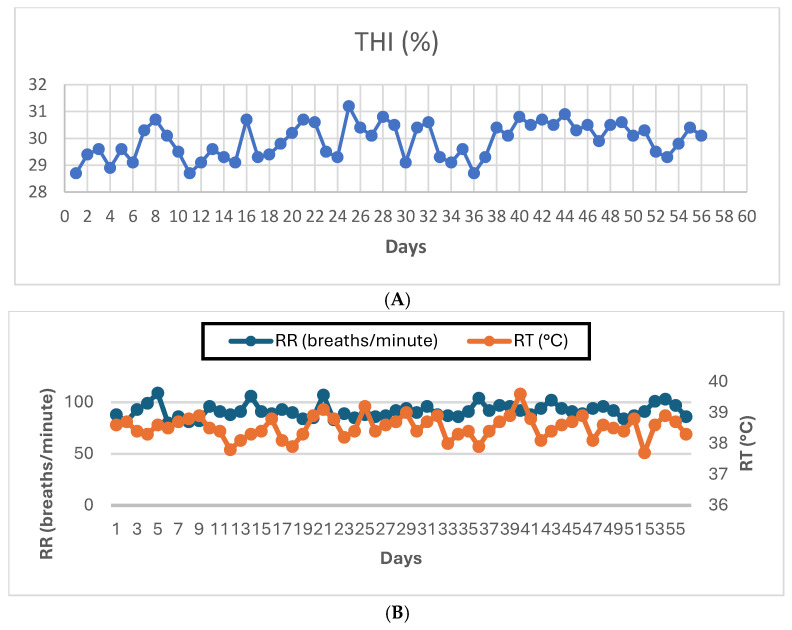
Heat Stress Indicators on Rabbits: The heat stress index ((**A**), THI), the respiratory rate ((**B**), RR), and the rectal temperature ((**B**), RT).

Figure 1 shows the extent of heat stress in rabbits. The heat stress index (THI) showed a significant increase, ranging between 28.7 and 31.2 °C throughout the experiment, indicating that the experimental rabbits were subjected to heat stress. In addition, the respiratory rate (RR, breaths/min) was high, ranging from 80 to 109 breaths/min. However, the rectal temperature (RT) ranged between 37.8 and 39.2 °C.

### 3.2. Growth and Carcass Characteristics

Table 4 summarizes the effects of ascorbic acid and the essential oil blend on the growth performance indicators and carcass characteristics of heat-stressed male rabbits. Rabbits receiving the combined treatment (MAO) exhibited a significant increase (*p* < 0.01) in final body weight compared with the control group, with values comparable to those observed in the EOB and ASA groups, indicating no additive effect of the combination treatment. Furthermore, the mortality rate decreased by 1.4–3.0% in the MAO-treated group compared to the other heat-stressed groups. In addition, a significant increase (*p* < 0.01) in carcass weight was observed in the groups receiving ASA, EOB, and MAO compared to the CON group. Interestingly, the groups supplemented with EOB and MAO showed the highest carcass weight. Although the kidneys, heart, and lungs were unaffected (*p* ≥ 0.05) by the experimental additions, the relative liver weight in rabbits fed ASA, EOB, and MAO was higher (*p* < 0.05) than that of the control group. Moreover, the relative weight of abdominal fat in rabbits fed EOB and MAO was lower (*p* < 0.05) than that of the ASA and control groups; however, it was even lower in the EOB- and MAO-treated groups. These findings indicate that the inclusion of essential oils, either alone (EOB) or in combination with ascorbic acid (MAO), produced the dominant effect on carcass traits.

### 3.3. Digestive Indicators

Table 5 presents the effects of ascorbic acid and essential oil blend supplementation on the digestive performance of heat-stressed rabbits. Trypsin (*p* < 0.01) and cellulase (*p* = 0.013) enzyme secretion increased in the EOB- and MAO-treated groups compared to the ASA and control groups; however, amylase enzyme secretion was unaffected. Dry matter digestion increased in the MAO-treated group (*p* < 0.01) compared to the ASA, EOB, and CON groups, indicating an additional improvement beyond individual supplementation (potential synergistic effect). Moreover, crude protein digestion increased (*p* < 0.05) in the EOB, ASA, and MAO groups compared with the CON group. Nevertheless, crude fiber digestion tended to increase (*p* < 0.05) in the EOB- and MAO-treated groups compared to the ASA and CON groups. Nevertheless, there was no difference in the groups’ digestion of NFE and ether extract (*p* ≥ 0.05).

### 3.4. Antioxidants Capacity

Table 6 presents the impact of ascorbic acid and essential oil blend supplementation on oxidative stability in the blood and seminal plasma of heat-stressed rabbits. Although MDA levels were lower in the EOB, MAO, and ASA groups (*p* < 0.01) than in the CON group, SOD (*p* < 0.01) and GPx (*p* = 0.004) enzyme activities were higher in the same groups than in the control group in blood plasma. Comparably, MDA levels decreased (*p* < 0.05) in conjunction with increased TAC (*p* < 0.05) and GSH (*p* < 0.01) enzyme activity in the EOB, MAO, and ASA groups compared to the CON group in seminal plasma. Interestingly, the combination treatment (MAO) consistently demonstrated a superior antioxidant response compared to the administration of EOB or ASA alone, indicating a strong synergistic effect under conditions of heat stress.

### 3.5. Biochemical Markers

**Figure 2:**
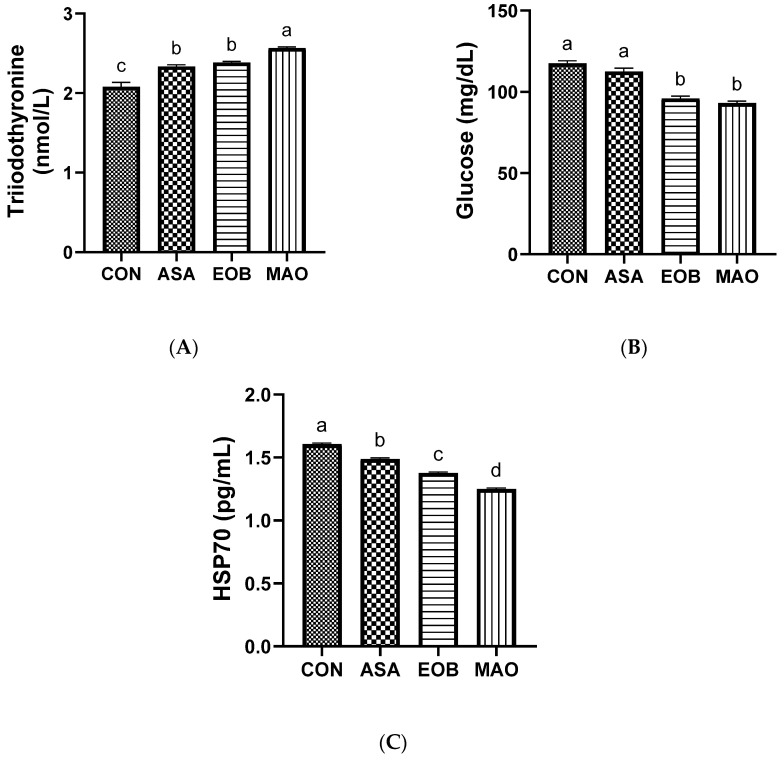
Triiodothyronine ((**A**), T3), glucose (**B**), and heat shock protein 70 ((**C**), HSP70) levels in heat-stressed rabbits fed ascorbic acid and essential oil blend. CON: group fed a basal diet; ASA: group fed a basal diet containing ascorbic acid; EOB: group fed a basal diet containing essential oil blend; MAO: group fed a basal diet containing ascorbic acid and essential oil blend. ^a–d^: Means within the same column with various superscripts are significantly different at the 5% level. Data are offered as mean ± SD.

Table 7 and Figure 2A–C illustrate the effects of ascorbic acid and essential oil blend supplementation on biochemical markers (lipid profile, liver and kidney function, and stress index) in heat-stressed rabbits. The lipid profile showed significant changes between the experimental treatments, with increased HDL levels (*p* < 0.05), while cholesterol (*p* < 0.01) and triglyceride (*p* < 0.05) levels decreased in the EOB- and MAO-treated groups compared with the ASA and CON groups. Furthermore, urea, creatinine, and AST levels were reduced (*p* < 0.05) in the EOB, MAO, and ASA groups compared with the control group. Moreover, total protein concentration was higher (*p* < 0.05) in the EOB- and MAO-treated groups than in the CON and ASA groups. Similarly, the experimental supplements positively affected stress markers. Glucose levels were reduced (*p* < 0.05) in the EOB- and MAO-treated groups compared to the ASA and CON groups, and heat shock protein 70 (HSP70) levels decreased (*p* < 0.05) in the EOB, MAO, and ASA groups compared with the CON group. Meanwhile, T3 levels increased (*p* < 0.05) in the EOB, MAO, and ASA groups compared with the CON group, with the highest level observed in the MAO group. LDL, ALB, and ALT levels were not affected by experimental supplementation (*p* ≥ 0.05). However, the results showed a synergistic effect of the compound on protein metabolism and stress modulation, while no such effect was observed in improving lipid metabolism.

### 3.6. Immunity

**Figure 3:**
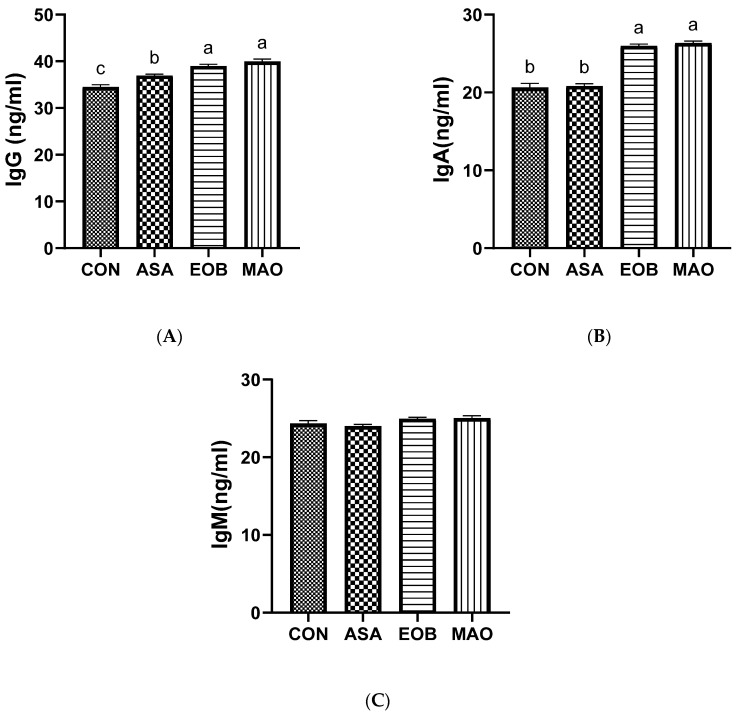
Immunoglobulin G ((**A**), IgG), immunoglobulin A ((**B**), IgA), and immunoglobulin M ((**C**), IgM) levels in heat-stressed rabbits fed ascorbic acid and essential oil blend. CON; group fed a basal diet, ASA; group fed a basal diet containing ascorbic acid, EOB; group fed a basal diet containing essential oil blend, MAO; group fed a basal diet containing ascorbic acid and essential oil blend. ^a–c^: At the 5% level, means in the same column with different superscripts differ significantly. Data are offered as mean ± SD.

**Figure 4:**
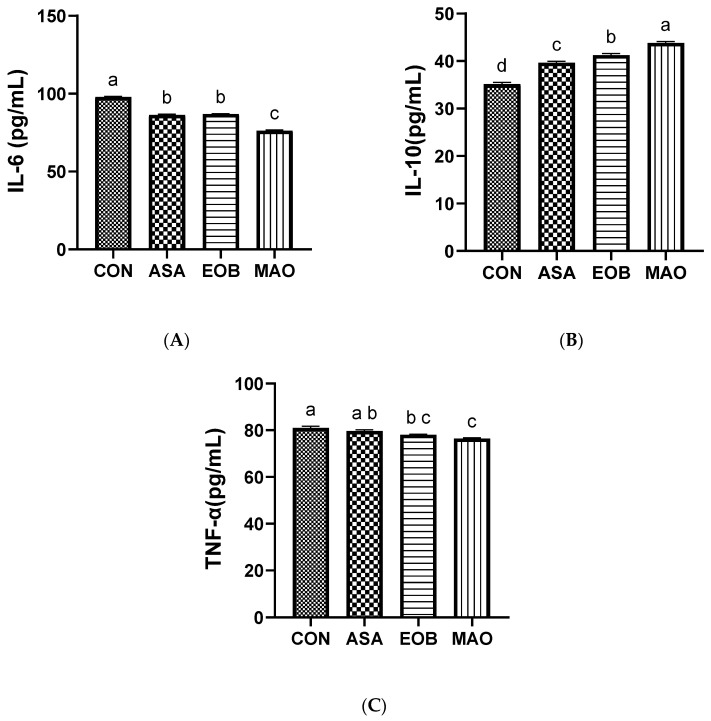
Interleukin 6 ((**A**), IL-6), interleukin 10 ((**B**), IL-10), and tumor necrosis factor-alpha ((**C**), TNF-α) levels in heat-stressed rabbits fed ascorbic acid and essential oil blend. CON: rabbits fed a basal diet; ASA: rabbits fed a basal diet containing ascorbic acid; EOB: rabbits fed a basal diet containing essential oil blend; MAO: rabbits fed a basal diet containing ascorbic acid and essential oil blend. ^a–d^: At the 5% level, means in the same column with different superscripts differ significantly. Data are offered as mean ± SD.

Figure 3A–C and Figure 4A–C illustrate the effects of adding ascorbic acid and an essential oil blend on immune response, including immunocytokines and immunoglobulins. IgA levels were higher (*p* < 0.05) in the EOB-and MAO-treated groups, whereas IgG levels were higher (*p* < 0.01) in the EOB, MAO, and ASA groups than in the CON group. Conversely, IgM levels tended to be higher (*p* < 0.05) in the EOB- and MAO-treated groups than in the ASA and CON groups. Moreover, IL-10 levels were higher (*p* < 0.01) in the EOB, MAO, and ASA groups than in the control group. Furthermore, IL-6 and TNF-α levels decreased (*p* < 0.05) in the EOB, MAO, and ASA groups compared with those in the CON group, with a significant decrease observed specifically in the MAO-treated group. It is noteworthy that the combination treatment (MAO) demonstrated a stronger effect on both IL-6 and IL-10, while this effect was not observed in the other immunological parameters.

### 3.7. Semen Quality

As shown in Table 8, ascorbic acid, essential oil blend, or their mixture improved semen quality in male rabbits. Sperm density, semen volume, sperm progressive motility, sperm vitality, and sperm normality increased (*p* < 0.01), whereas sperm abnormality decreased (*p* < 0.01) in the treated groups compared to the CON group. Additionally, the pH of semen tended to be higher (*p* < 0.05) in the EOB- and MAO-treated groups than in the other groups. Interestingly, the highest values for sperm density, semen volume, sperm progressive motility, and sperm vitality were recorded in rabbits that received MAO, that the combination treatment (MAO) had a synergistic effect on semen quality.

### 3.8. Microbial Enumeration

Ascorbic acid and essential oil blend supplements exerted strong modulatory effects on intestinal microbiota in heat-stressed rabbits (Table 9). *C. perfringens* population was lower (*p* < 0.01) in the EOB- and MAO-treated groups than in the other groups, whereas the population of *E. coli* was lower (*p* < 0.01) in rabbits fed ASA, EOB, and MAO groups than in the control group. Furthermore, total coliforms tended to be lower (*p* < 0.05) in the EOB- and MAO-treated groups than in rabbits fed ASA and control groups. Moreover, *Lactobacillus* count was higher (*p* < 0.01) in the EOB- and MAO-treated groups than in the other groups. The counts of T. bacteria and *Enterococcus* were not affected by the experimental treatments (*p* ≥ 0.05). This suggests that the combination treatment had no additional effect compared to the individual additions on the microbial count.

### 3.9. Gene Expression

**Figure 5:**
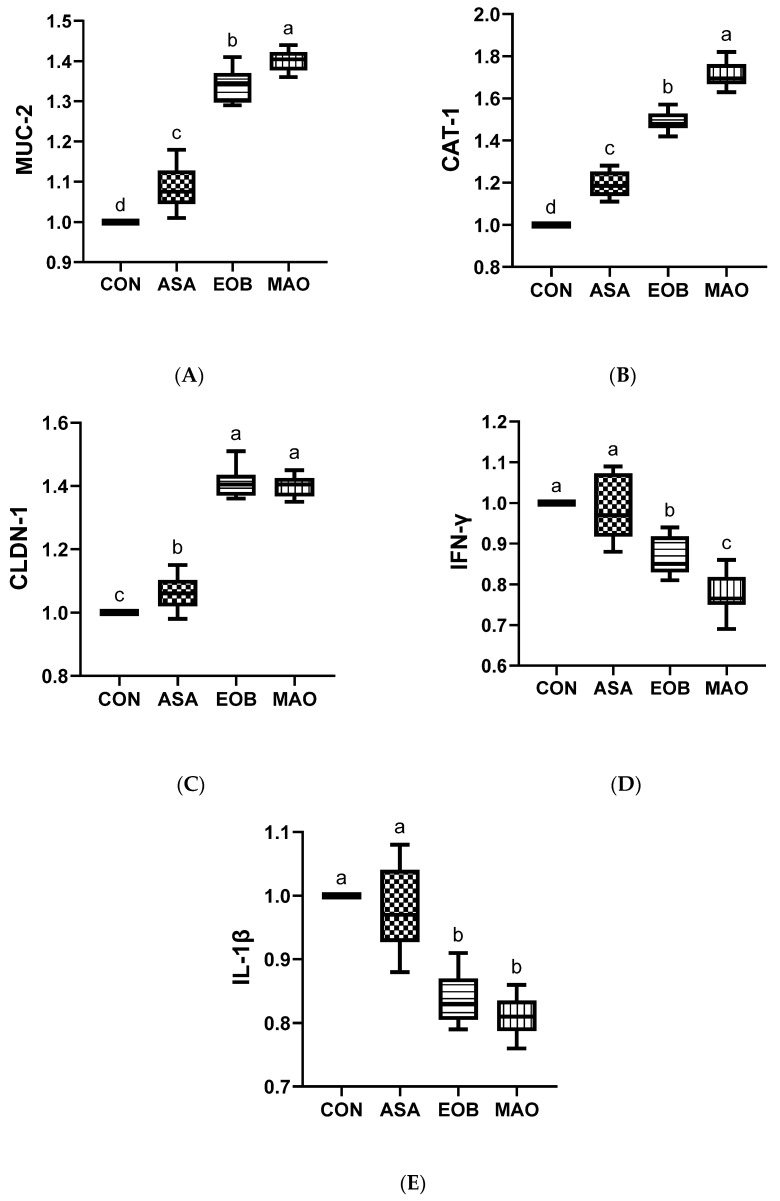
Mucin-2 ((**A**), MUC-2), cationic amino acid transporter-1 ((**B**), CAT-1), claudin-1 ((**C**), CLDN-1), interleukin-1β ((**E**), IL-1β), and interferon-gamma ((**D**), IFN-γ) levels mRNA expression in heat-stressed rabbits fed ascorbic acid and essential oil blend. CON: rabbits fed a basal diet; ASA: rabbits fed a basal diet including ascorbic acid; EOB: rabbits fed a basal diet including essential oil blend; MAO: rabbits fed a basal diet including ascorbic acid and essential oil blend. ^a–d^: At the 5% level, means in the same column with different superscripts differ significantly. Data are offered as mean ± SD.

Ascorbic acid and essential oil blend supplementation affected gene expression modulation (Figure 5A–E). Dietary treatments upregulated the MUC-2, CAT-1, and CLDN-1 genes compared to the CON group. The highest expression of the MUC-2 and CAT-1 genes was observed (*p* < 0.01) in the MAO-treated group compared to the other groups; however, the highest expression of the CLDN-1 gene was observed in the MAO- and EOB-treated groups (*p* < 0.05) compared to the other groups. Additionally, the expression of the IFN-γ and IL-1β genes was decreased (*p* < 0.05) in the MAO- and EOB-treated groups compared to the other groups. This suggests that the combination treatment had a stronger regulatory effect on the expression levels of the MUC-2, CAT-1, and IFN-γ genes compared to the individual additives.

## 4. Discussion

Despite increasing interest in finding an effective strategy to reduce heat stress damage, rabbit breeders continue to suffer [10,11]. Our study data are consistent with this and showed that rabbits were exposed to heat stress through increased respiratory rates (RRs), accompanied by higher values of the temperature and humidity index (THI). Interestingly, the use of nutritional supplements as a nutritional strategy has proven effective in alleviating the negative effects of heat stress [13,14,18,19]. Therefore, the effect of the dietary combination of ascorbic acid and essential oil blend supplements was investigated to support physiological performance and general health. The results of the study demonstrated positive effects, promoting growth, immune response, redox status, intestinal health, and sperm quality.

The present results demonstrate that dietary supplementation with ascorbic acid and an essential oil blend effectively mitigated the adverse effects of heat stress in rabbits, as evidenced by improved growth performance and increased carcass weights. The improvement in the performance of heat-stressed rabbits that were fed ascorbic acid and essential oil supplements could be attributed to the effects of these compounds as antioxidants and anti-inflammatories [24,42], as well as the role of essential oils as antimicrobials [26,37]. However, other studies have not shown any differences compared to the control group for the addition of supplements [43,44], and this discrepancy in results may be due to several differences related to the type, purity, dosage, and form of essential oils [45]. Despite that, many reports have shown the positive effects of ascorbic acid supplements, such as neutralizing free radicals, reducing inflammation resulting from stress, and protecting cell membranes, thus preserving the structure of the intestines and immune systems [19], which enhances the metabolism of proteins, carbohydrates, and fats, and thus improves the absorption of nutrients [42,43]. Concurrently, the ingestion of essential oils improves productive performance under heat stress through several mechanisms, including enhancing metabolic adaptation, modifying microbial content [21,24], and immunomodulation [27], as well as enhancing the secretion of digestive enzymes [22,31], which enhances the overall performance of stressed rabbits. In addition, this study found that the addition of the mixture led to an increase in relative liver weight and a decrease in abdominal fat accumulation in heat-stressed rabbits, as also noted by Elbaz et al. [31]. Conversely, other reports indicate that the addition of essential oils to feed did not affect fat percentages in the abdomen, liver, lymphatic organs, or gizzard [45,46,47]. The differences in responses to added essential oils between studies can be attributed to various factors, including health conditions, environmental conditions, feed composition, and the type and dosage of the added essential oils [45,48]. The improvement in liver weight in heat-stressed rabbits in the current study can be explained by the effect of nutritional supplementation in enhancing antioxidant defense systems during stress exposure, thereby reducing reactive oxygen and increasing internal oxidative enzymes [19,49], and reducing oxidative damage, which may promote the stability of intestinal and liver functions. Therefore, these results indicate that essential oils and ascorbic acid were the primary drivers of improved growth and carcass characteristics, while their combination maintained these effects without enhancing them in rabbits during heat stress.

Consistent with the observed growth performance, the results of the current study indicate that the combined supplementation of ascorbic acid and an essential oil blend may contribute to enhancing digestive function, as evidenced by improved nutrient digestion and stimulated digestive enzyme production. Likewise, many reports indicate that ascorbic acid and essential oil supplements have positive effects on nutrient digestion and increased digestive enzyme secretion [17,37]. The enhancing effect of ascorbic acid on digestive physiology may be attributed to reducing oxidative damage to cells and stabilizing cell membranes, which maintains the integrity of the intestinal villi and their surface area [50,51], enhancing nutrient absorption. Meanwhile, essential oils have proven effective in promoting digestive secretions through the action of their biologically active compounds in enhancing bile flow and increasing pancreatic enzyme secretion [31,52], thus improving nutrient digestion. Furthermore, the antimicrobial properties of these compounds help regulate intestinal microbiota [52], reduce intestinal inflammation [24], and improve nutrient utilization efficiency. High temperatures also disrupt fat metabolism in rabbits, leading to fat accumulation [8], which is in accordance with our findings. Meanwhile, supplementation with ascorbic acid and essential oil may have helped regulate fat metabolism in heat-stressed rabbits, resulting in reduced abdominal fat. Overall, these results indicate that the combined group exhibited enhanced digestive efficiency, demonstrating a synergistic interaction between essential oils and ascorbic acid that transcends the effects of either component alone under the conditions of this study.

Heat stress disrupts the oxidation–reduction balance, and a large number of reactive oxygen species (ROS) are released into the rabbits’ blood and seminal plasma, subsequent lipid peroxidation, which impairs cellular components and negatively affects physiological performance and semen quality [8,9,12,53]. Antioxidant enzymes, which have high oxidative activity and eliminate hydrogen peroxide and oxygen ions, are the first line of defense [2,13,17], through a synergistic effect in the body to prevent cell damage and maintain physiological functions. In this context, the results of the current study showed that adding a mixture of ascorbic acid and an essential oil blend to the diet demonstrated an antioxidant effect through increased blood SOD and GPx, increased TAC and GSH in seminal plasma, as well as a significant decrease in MDA levels within the blood plasma and seminal plasma, which is consistent with many previous reports [24,26,31,34]. Ascorbic acid participates in the removal of free radicals resulting from cell peroxidation in the body [46,54], thus protecting tissue cells from oxidative damage [55,56]. This is supported by the reduction in MDA levels, suggesting decreased membrane lipid oxidation and improved redox balance. Additionally, essential oils directly improve the oxidative status of rabbits suffering from heat stress through several mechanisms associated with the active compounds [24,26,57,58], including the direct scavenging of reactive oxygen species and hydroxyl radicals [8], and the activation of cellular signaling pathways and endogenous antioxidant enzymes through the Nrf2 gene signaling pathways [59,60], which reduces lipid peroxidation. The oxidation state results support the idea that the combined supplementation creates a synergistic effect between the two components, improving the redox balance under heat stress conditions more effectively than individual enhancements.

Oxidative stress resulting from heat stress leads to increased fat oxidation [57] and may contribute to impaired liver and kidney functions through increased free-radical production [37] and disorders in the rabbit’s internal oxidative system [35]. In the current study, the dietary combination of ascorbic acid and an essential oil blend reduced cholesterol, triglyceride, AST, urea, and creatinine levels, while increasing HDL-C and total protein levels. Studies have shown that adding essential oils improves blood biochemistry, reduces lipid peroxidation [60,61], and enhances antioxidant capacity [35], which is attributed to the role of the oil components in maintaining the integrity of kidney and liver cells, the main factories of lipid metabolism [61], as evidenced by the decrease in AST, urea, and creatinine levels. Additionally, essential oils improve fat metabolism and digestion [62] by inhibiting the activity of enzymes involved in cholesterol metabolism and synthesis, including 3-hydroxy-3-methylglutaryl-CoA reductase (HMGCR) and cholesterol 7-alpha-hydroxylase (CYP7A1) [60]. Ascorbic acid may also contribute to protecting kidney and liver tissue cells from oxidative damage [18,24] by participating in redox reactions and limit cell peroxidation in the body by removing free radicals [32,56] resulting from oxidative stress during heat stress [63].

Blood glucose and triiodothyronine (T3) levels, and heat shock protein 70 (HSP70), are among the most sensitive indicators of heat stress as a cellular protective response [8]. Our results showed that adding ascorbic acid and an essential oil blend to the diet supported rabbits’ stress resistance by regulating metabolism through lower glucose and HSP70 levels and increased blood T3 levels. These changes indicate a more stable metabolic state and reduced cellular stress in supplemented rabbits [64,65]. The decline in HSP70 levels could indicate a lower degree of cellular stress, as HSP70 is typically upregulated in response to protein damage and oxidative challenges [43,64]. This observation may suggest a potential role of the dietary treatments in supporting antioxidant defenses and limiting oxidative damage at the cellular level, thus improving protein synthesis and maintaining cell integrity [66,67]. Additionally, the increase in T3 levels suggests improved thyroid activity and metabolic balance, which are often suppressed under heat stress conditions [10,67,68]. This restoration may be associated with reduced oxidative damage to endocrine tissues and improved physiological regulation [31,67,69]. In contrast, the reduction in blood glucose suggests improved regulation of energy metabolism under heat stress and more efficient utilization of available energy substrates [17,67]. These results indicate that the essential oils, both individually and in combination, primarily affected lipid metabolism, while the combined treatment had a more pronounced effect on protein metabolism and reduced stress markers, which could contribute to reduced oxidative damage and improved metabolic stability, cellular integrity, and nutrient utilization in heat-stressed rabbits.

The findings of the current study revealed that the control group had much higher levels of inflammatory cytokines and significantly lower levels of immunoglobulins. These findings may be attributed to heat-stress induced inflammation in immune organs and oxidative damage that impairs immune cell function, which suppresses immune responses and alters the expression of inflammatory cytokines (e.g., TNF-α and IL-6) in the small intestine and plasma; in addition, heat stress leads to elevated cortisol levels, which inhibit antibody production [70,71,72]. However, feeding rabbits a mixture of ascorbic acid and essential oil blend enhanced the immune response, increasing levels of IgA, IgM, and IgG, and IL-10 while decreasing IL-6 and TNF-α levels. These changes may indicate a shift toward a more balanced immune profile in supplemented rabbits. Such effects could be related to the antioxidant properties of ascorbic acid and phenolic compounds present in essential oils, which may reduce reactive oxygen species (ROS) and support endogenous antioxidant systems [50,73,74]. In turn, this enhances antibody production and modulates cytokine balance [42,74,75], thus supporting and stabilizing the immune system. Furthermore, essential oils play a role in reducing inflammatory response disturbances [22,76] and improving intestinal integrity [24,76] and mucosal immunity, thereby enhancing the absorption of nutrients necessary for immunoglobulin A (IgA) synthesis [60,63]. These observations suggest that taking the combined supplements showed a stronger effect on both pro-inflammatory and anti-inflammatory cytokines, which may contribute to improving the immune system efficiency under conditions of heat stress.

The plasma membrane of rabbit sperm is rich in unsaturated fatty acids, which may render it more susceptible to free radical attacks during heat stress [2]. This can promote lipid peroxidation, potentially impairing DNA stability and membrane integrity, and thereby reducing sperm motility and fertilization capacity [77]. This improvement is attributed to the synergistic effect of ascorbic acid and an essential oil blend in enhancing antioxidant defenses and improving endocrine regulation under heat-stress conditions [70,73]. This is demonstrated by the fact that the bioactive compounds in essential oils enhance the activity of endogenous antioxidant enzymes [60,63]. Additionally, ascorbic acid acts as a potent free radical scavenger [34,46]. Such actions may help limit lipid peroxidation and preserve sperm membrane function [34,74]. Furthermore, supplementation reduces oxidative and inflammatory damage [21,26,67], protecting testicular tissue and supporting sperm formation and integrity [2,77]. This, in turn, may contribute to increased sperm motility, viability, and concentration, while reducing sperm abnormalities. The noteworthy improvement in all semen traits in the combined supplementation group provides strong evidence of a greater-than-individual effect, highlighting the role of integrated antioxidant protection in optimizing reproductive performance. Nevertheless, further studies are warranted to investigate the long-term effects of this mixture on reproductive efficiency, offspring viability, and overall progeny health.

Maintaining intestinal integrity is essential for improving nutritional efficiency, systemic immune balance, and growth performance, especially during heat stress, which may negatively affect intestinal permeability, inflammatory status, and microbial composition [23,31,37]. The data from the current study showed a modulating effect of ascorbic acid-essential oil mixture or essential oils supplementation on gut microbiota, as evidenced by reduced pathogenic bacterial counts. These results are consistent with many previous reports demonstrating the antimicrobial properties of essential oils and ascorbic acid [78,79,80,81,82]. This effect is attributed to the active compounds in essential oils exhibiting selective antimicrobial effects against pathogenic bacteria [31,60] by weakening metabolic processes and disrupting cell membranes [83], while ascorbic acid reduces the pH of the intestinal environment, thus limiting the proliferation of pathogens [71,73], demonstrating an effect in improving microbial balance and intestinal health. Therefore, essential oils, whether used alone or in a mixture, are likely to exert antimicrobial effects, as the combination supplementation maintained this effect without addition, which may promote gut health by suppressing pathogenic microorganisms and thus reducing toxins derived from the pathogenic microbiome and local inflammation, both of which are typically increased during heat stress.

Profound molecular changes occur in heat-stressed rabbits in response to heat stress, which alters the expression of genes associated with stress response, antioxidant defense, metabolism, immune regulation, and genes responsible for intestinal barrier function, in addition to increasing the expression of pro-inflammatory cytokines, negatively affecting growth rate, feeding efficiency, immune system efficiency, and intestinal integrity. Our results showed that rabbits receiving a mixture of ascorbic acid and an essential oil blend enhanced intestinal integrity by increasing the expression levels of CAT-1, CLDN-1, and MUC-2 genes, whereas the expression levels of IL-1β and IFN-γ decreased. Similarly, many reports have indicated the positive effects of ascorbic acid or essential oil supplements in regulating genes associated with gut health in animals under heat stress [2,9,19]. A noteworthy decrease in IL-1β and IFN-γ gene expression levels in rabbits treated with ascorbic acid and essential oils may be attributed to their antioxidant and anti-inflammatory properties [22,26]. These properties include potent reactive oxygen species scavenger activity and increased activity of oxidative stress-related enzymes [34,84,85], thereby reducing oxidative stress and inflammation induced by heat stress. Concurrently, the addition of ascorbic acid and essential oils may promote the restoration of CAT-1, CLDN-1, and MUC-2 expression through several mechanisms, including the suppression of inflammatory and oxidative damage [86,87]. These beneficial effects mitigate the cascade of inflammatory reactions and maintain epithelial tissue stability, protecting cells and tight junction proteins and enhancing mucus thickness [85,88], thereby supporting intestinal integrity. Based on these effects, the combined supplementation of ascorbic acid and essential oils may modulate gene expression during heat stress conditions, which may contribute to improved intestinal function, immune system efficiency, and nutrient absorption efficiency in heat-stressed rabbits.

Despite these promising results from the current study, some limitations include the small experimental unit of data from subsamples (such as blood, microbiome, and gene expression data) and the fact that the essential oil blend was not fully characterized chemically, limiting the interpretation of each compound. Therefore, future studies using comprehensive structural analyses of the essential oil blend (such as GC-MS) are needed, along with an evaluation of the supplement’s effects under a variety of experimental conditions.

## 5. Conclusions

Dietary supplementation with a combination of the essential oil blend and ascorbic acid was associated with improvements in several physiological, reproductive, and productive parameters in heat-stressed rabbits. These effects included enhancements in growth performance, antioxidant status, immune-related indicators, and sperm quality, as well as improvements in selected intestinal health parameters. These findings indicate that this combined nutritional approach may contribute to alleviating some negative effects of heat stress. However, further studies are required to confirm the underlying mechanisms and validate these findings under different production conditions.

## Figures and Tables

**Table 1:** Constituents and chemical assessment of the basal diet.

Ingredient	%
Berseem hay	34.1
Soybean meal (44%)	16.0
Yellow corn	10.0
Wheat bran	17.5
Barley grains	15.0
Sunflower meal	2.00
Molasses	3.00
Di-Calcium phosphate	1.60
Salt (NaCl)	0.50
Vit. & Min. Premix *	0.30
**Chemical composition**	
Digestible Energy (Kcal/Kg)	2496
Crude protein (%)	16.7
Crude fiber (%)	12.6
Calcium (%)	1.10
Phosphorus (%)	0.60

* Each 1 kg of vitamin-mineral premix contained: 5460 mg of phylloquinone, 5640 mg of thiamine, 14,560 mg of riboflavin, 13,130 mg of DL-3-tocopheryl acetate, 27,300 mg of Ca-D-pantothenate, 12,000 mg of manganese, 3640 mg of folic acid, 7350 mg of pyridoxine, 10,920 mg of niacin, 2370 mg of D-biotin, 900 mg of zinc, 12,500 mg of iodine, 3000 mg of selenium, 29.12 mg of cobalamin, 160 mg of copper, and 40,000 mg of ferrous.

**Table 2:** Incubation conditions for microbiological analysis in cecum contents.

Microbial Analysis	Temp. (°C)	Period	Growth Medium (Agar)	References
*Total bacterial count*	37	24–48 h	Plate Count	[38]
*Lactobacillus*	37	48 h	de Man, Rogosa, and Sharpe	[39]
*Enterococcus*	37	24 h	Bile esculin azide	[38]
*Escherichia coli*	37	24 h	Eosin methylene blue	[38]
*Total coliforms*	37	24 h	MacConkey	[38]
*Clostridium perfringens*	37	24 h	Tryptose Sulfite Cycloserine	[40]

**Table 3:** Primer sequences of target genes analyzed.

Target Genes	Forward 5′ → 3′	Reverse 5′ → 3′
MUC-2	5′-TATACCGCAAGCAGCCAGGT-3′	5′-GCAAGCAGGACACAGACCAG-3′
CAT-1	5′-CCAGTCTATTAGGTTCCATGTTCC-3′	5′-CGATTATTGGCGTTTTGGTC-3′
CLDN-1	5′-GGAGCAAAAGATGCGGATGG-3′	5′-AATTGACAGGGGTCAAAGGGT-3′
IL-1β	5′-TGTCAGTCGTTGTGGCTCTG-3′	5′-AGTCATCCCAGGTGTTGCAG-3′
IFN-γ	5′-TTCTTCAGCCTCACTCTCTCC-3′	5′-TGTTGTCACTCTCCTCTTTCC-3′
GAPDH	5′-TGTTTGTGATGGGCGTGAA-3′	5′-CCTCCACAATGCCGAAGT-3′

MUC-2, mucin-2; CLDN-1, claudin-1; CAT-1, cationic amino acid transporter-1; IL-1β, interleukin-1β; and IFN-γ, interferon-gamma.

**Table 4:** Effects of ascorbic acid and essential oil blend on growth and carcass index of male rabbits.

	Parameter	CON	ASA	EOB	MAO	*p* Value
Growth performance	IBW, g	2961 ± 16.7	2938 ± 13.3	2954 ± 14.2	2947 ± 13.6	0.415
	FBW, g	2887 ± 11.8 ^b^	2963 ± 12.9 ^a^	3001 ± 10.0 ^a^	2995 ± 11.5 ^a^	0.001
	FI, g	88.9 ± 3.52	89.3 ± 2.91	90.2 ± 3.04	89.4 ± 2.83	0.266
	MOR, %	11.4	8.57	5.71	5.71	-
Carcass Characteristics	Pre-slaughter, g	2873 ± 16.7 ^b^	2951 ± 13.4 ^a^	2972 ± 14.1 ^a^	2981 ± 13.8 ^a^	0.001
	CW, %	58.4 ± 4.82 ^c^	59.7 ± 3.92 ^b^	61.2 ± 3.35 ^a^	61.5 ± 3.74 ^a^	0.001
	Liver, %	3.06 ± 0.17 ^b^	3.42 ± 0.13 ^a^	3.39 ± 0.15 ^a^	3.44 ± 0.12 ^a^	0.020
	Kidney, %	0.78 ± 0.08	0.75 ± 0.02	0.78 ± 0.4	0.76 ± 0.03	0.183
	Heart, %	0.30 ± 0.02	0.29 ± 0.04	0.31 ± 0.03	0.30 ± 0.02	0.257
	Lungs, %	0.66 ± 0.05	0.65 ± 0.07	0.67 ± 0.01	0.68 ± 0.01	0.115
	A. Fat, %	2.03 ± 0.18 ^a^	1.87 ± 0.09 ^a^	1.06 ± 0.11 ^b^	1.11 ± 0.20 ^b^	0.004

CON: group fed a basal diet; ASA: group fed a basal diet containing ascorbic acid; EOB: group fed a basal diet containing essential oil blend; MAO: group fed a basal diet containing ascorbic acid and essential oil blend. FI: feed intake; IBW: initial body weight; FBW: final body weight; CW: carcass weight; MOR: mortality rate; A. fat: abdominal fat. Distinct superscript letters (*p* < 0.05) indicate means that differ significantly within the same row. Values are presented as mean ± SD.

**Table 5:** Effects of ascorbic acid and essential oil blend on digestive enzyme activity and digestibility of male rabbits.

	Parameter	CON	ASA	EOB	MAO	*p* Value
Digestibility	Amylase, U/g	2.83 ± 1.74	2.77 ± 1.52	2.86 ± 1.90	2.91 ± 1.33	0.107
	Trypsin, KU/mg	17.6 ± 2.55 ^b^	18.1 ± 2.71 ^b^	20.3 ± 2.36 ^a^	20.6 ± 2.42 ^a^	0.001
	Cellulase, U/g	2.14 ± 0.37 ^b^	2.09 ± 0.56 ^b^	2.35 ± 0.39 ^a^	2.41 ± 0.43 ^a^	0.013
Digestive enzyme activity	Dry matter, %	61.7 ± 2.06 ^c^	63.4 ± 2.17 ^bc^	64.2 ± 1.95 ^b^	65.8 ± 2.01 ^a^	0.001
	Crude protein, %	70.3 ± 1.88 ^c^	71.3 ± 1.92 ^b^	73.6 ± 1.71 ^a^	74.0 ± 1.82 ^a^	0.001
	Crude fiber, %	45.1 ± 0.57 ^b^	45.4 ± 0.61 ^b^	46.1 ± 0.44 ^ab^	47.2 ± 0.53 ^a^	0.006
	Ether extract, %	80.2 ± 4.06	79.6 ± 4.11	81.1 ± 3.58	80.8 ± 3.75	0.228
	NFE, %	54.9 ± 2.12	54.3 ± 1.99	55.3 ± 2.04	55.6 ± 2.17	0.173

CON: group fed a basal diet; ASA: group fed a basal diet containing ascorbic acid: EOB: group fed a basal diet containing essential oil blend; MAO: group fed a basal diet containing ascorbic acid and essential oil blend. NFE: nitrogen-free extract. Distinct superscript letters (*p* < 0.05) indicate means that differ significantly within the same row. Values are presented as mean ± SD.

**Table 6:** Effects of ascorbic acid and essential oil blend on antioxidant capacity in blood and seminal of male rabbits.

	Parameter	CON	ASA	EOB	MAO	*p* Value
Blood plasma	MDA, nmol/mL	2.61 ± 0.84 ^a^	2.08 ± 0.99 ^b^	1.75 ± 0.76 ^bc^	1.31 ± 0.88 ^c^	0.001
	SOD, U/mL	33.7 ± 1.32 ^d^	38.2 ± 1.07 ^b^	36.4 ± 1.28 ^c^	40.6 ± 1.53 ^a^	0.001
	GPx, U/L	24.1 ± 0.48 ^c^	26.8 ± 0.66 ^b^	27.1 ± 0.51 ^b^	29.3 ± 0.54 ^a^	0.004
Seminal plasma	MDA, nmol/mL	4.91 ± 1.17 ^a^	3.54 ± 1.09 ^b^	3.48 ± 1.31 ^b^	2.86 ± 1.13 ^c^	0.001
	TAC, nmol/mL	17.5 ± 0.66 ^c^	20.3 ± 0.72 ^b^	19.6 ± 0.48 ^b^	23.7 ± 0.65 ^a^	0.018
	GSH, lmol/mL	0.38 ± 0.08 ^c^	0.57 ± 0.05 ^ab^	0.50 ± 0.09 ^b^	0.62 ± 0.12 ^a^	0.001

CON: group fed a basal diet; ASA: group fed a basal diet containing ascorbic acid; EOB: group fed a basal diet containing essential oil blend; MAO: group fed a basal diet containing ascorbic acid and essential oil blend. MDA: malondialdehyde; TAC: total antioxidant capacity; SOD: superoxide dismutase; GSH: reduced glutathione; GPx: glutathione peroxidase. Distinct superscript letters (*p* < 0.05) indicate means that differ significantly within the same row. Values are presented as mean ± SD.

**Table 7:** Effects of ascorbic acid and essential oil blend on the liver and kidney function and lipid profile of male rabbits.

	Parameter	CON	ASA	EOB	MAO	*p* Value
Lipid profile	CHO, mg/dL	104 ± 2.54 ^a^	105 ± 2.16 ^a^	89.1 ± 2.37 ^b^	88.7 ± 2.28 ^b^	0.001
	TRI, mg/dL	67.3 ± 1.22 ^a^	68.0 ± 1.19 ^a^	60.3 ± 1.42 ^b^	60.8 ± 1.27 ^b^	0.003
	HDL, mg/dL	25.4 ± 0.96 ^b^	26.1 ± 0.71 ^ab^	26.8 ± 0.59 ^a^	27.1 ± 0.84 ^a^	0.010
	LDL, mg/dL	20.7 ± 1.25	21.2 ± 1.37	19.6 ± 1.08	20.3 ± 1.21	0.097
Liver and kidney function	T.P, g/dL	6.35 ± 0.51 ^c^	6.41 ± 0.48 ^c^	6.78 ± 0.43 ^b^	7.11 ± 0.46 ^a^	0.001
	ALB, g/dL	3.02 ± 0.11	3.05 ± 0.15	2.97 ± 0.20	3.07 ± 0.14	0.182
	CRE, mg/dL	1.28 ± 0.08 ^a^	1.16 ± 0.06 ^b^	1.03 ± 0.10 ^bc^	0.92 ± 0.09 ^c^	0.001
	Urea, mg/dL	41.3 ± 1.94 ^a^	37.5 ± 2.01 ^b^	33.8 ± 1.88 ^c^	32.7 ± 1.97 ^c^	0.001
	AST, U/L	56.1 ± 2.33 ^a^	53.4 ± 1.87 ^b^	51.3 ± 2.05 ^c^	50.9 ± 2.13 ^c^	0.002
	ALT, U/L	13.8 ± 1.08	14.3 ± 1.32	14.1 ± 0.97	14.2 ± 1.14	0.311

CON: group fed a basal diet; ASA: group fed a basal diet containing ascorbic acid; EOB: group fed a basal diet containing essential oil blend; MAO: group fed a basal diet containing ascorbic acid and essential oil blend. T.P: total protein; ALB: Albumin; AST: aspartate aminotransferase; ALT: alanine aminotransferase; HDL: high-density lipoprotein; LDL: low-density lipoprotein; CHO: cholesterol; TRI: triglycerides; CRE: creatinine. Distinct superscript letters (*p* < 0.05) indicate means that differ significantly within the same row. Values are presented as mean ± SD.

**Table 8:** Effects of ascorbic acid and essential oil blend on semen quality of male rabbits.

Parameter	CON	ASA	EOB	MAO	*p* Value
Sperm density, 10^8^/mL	3.51 ± 0.85 ^c^	4.04 ± 0.79 ^b^	4.15 ± 0.91 ^b^	5.80 ± 0.82 ^a^	0.001
Semen pH	6.92 ± 1.75 ^b^	7.05 ± 2.67 ^ab^	7.18 ± 2.01 ^a^	7.11 ± 1.68 ^a^	0.040
Semen volume, mL	0.53 ± 0.07 ^c^	0.74 ± 0.05 ^b^	0.72 ± 0.06 ^b^	0.86 ± 0.07 ^a^	0.001
Sperm progressive motility, %	76.2 ± 8.11 ^c^	79.5 ± 6.21 ^b^	80.2 ± 7.05 ^b^	82.3 ± 8.37 ^a^	0.001
Sperm vitality, %	62.1 ± 5.82 ^d^	67.5 ± 3.74 ^c^	72.7 ± 4.68 ^b^	76.4 ± 3.91 ^a^	0.001
Sperm normality, %	48.3 ± 5.77 ^c^	59.8 ± 4.01 ^b^	71.5 ± 6.67 ^a^	73.6 ± 4.94 ^a^	0.001
Sperm abnormality, %	51.7 ± 5.41 ^a^	40.2 ± 4.22 ^b^	28.5 ± 6.35 ^c^	26.4 ± 3.82 ^c^	0.001

CON: group fed a basal diet; ASA: group fed a basal diet containing ascorbic acid; EOB: group fed a basal diet containing essential oil blend; MAO: group rabbits fed a basal diet containing ascorbic acid and essential oil blend. Distinct superscript letters (*p* < 0.05) indicate means that differ significantly within the same row. Values are presented as mean ± SD.

**Table 9:** Effects of ascorbic acid and essential oil blend on cecum microbial count (Log10 CFU g^−1^) of male rabbits.

Parameter	CON	ASA	EOB	MAO	*p* Value
*T. bacterial count*	17.6 ± 5.18	17.8 ± 4.67	18.1 ± 4.92	17.7 ± 4.73	0.221
*Enterococcus*	6.85 ± 1.44	6.92 ± 1.32	6.78 ± 1.41	6.81 ± 1.36	0.509
*Total Coliforms*	5.21 ± 2.07 ^a^	5.12 ± 1.95 ^ab^	4.91 ± 1.82 ^b^	4.89 ± 2.14 ^b^	0.013
*E. coli*	4.27 ± 1.28 ^a^	3.88 ± 1.33 ^b^	2.75 ± 1.41 ^c^	2.61 ± 1.09 ^c^	0.001
*C. perfringens*	3.69 ± 0.63 ^a^	3.54 ± 0.30 ^a^	2.64 ± 0.51 ^b^	2.09 ± 0.46 ^c^	0.001
*Lactobacillus*	4.38 ± 0.97 ^b^	4.51 ± 0.88 ^b^	6.23 ± 0.72 ^a^	6.42 ± 0.85 ^a^	0.001

CON: group fed a basal diet; ASA: group fed a basal diet including ascorbic acid; EOB: group fed a basal diet including essential oil blend; MAO: group fed a basal diet including ascorbic acid and essential oil blend. T. bacterial count, total bacterial count; *C. perfringens, Clostridium perfringens*; *E. coli, Escherichia coli*. Distinct superscript letters (*p* < 0.05) indicate means that differ significantly within the same row. Microbial count data were log_10_-transformed before statistical analysis to reduce heterogeneity of variance and achieve normality; statistical comparisons were performed on transformed data. Data are presented as mean ± SD.

## Data Availability

The original contributions presented in this study are included in the article. Further inquiries can be directed to the corresponding authors.

## Abbreviations

The following abbreviations are used in this manuscript:

A. fat	Abdominal fat
ALT	Alanine aminotransferase
ASA	Rabbits fed a basal diet including ascorbic acid
AST	Aspartate aminotransferase
C. perfringens	Clostridium perfringens
CAT-1	Cationic amino acid transporter-1
CLDN-1	Claudin-1
CON	Rabbits fed a basal diet
CW	Carcass weight
E. coli	Escherichia coli
EOB	Rabbits fed a basal diet including essential oil blend
FBW	Final body weight
FI	Feed intake
GPx	Glutathione peroxidase
GSH	Reduced glutathione
HDL	High-density lipoprotein
IBW	Initial body weight
IFN-γ	Interferon-gamma
IL-10	Interleukin 10
IL-1β	Interleu-kin-1β
LDL	low-density lipoprotein
MAO	Rabbits fed a basal diet including ascorbic acid and essential oil mixture
MUC-2	Mucin-2
NFE	Nitrogen-free extract
SOD	Superoxide dismutase
TAC	Total antioxidant capacity
TNF-α	Tumor necrosis factor-alpha

## References

[1] Imam M.A.A., Dorina M., Mohamed S., Ayman A., Monica M. (2020). Rabbits meat production in Egypt and its impact on food security, small holders income and economy. Agric. Sci. Technol..

[2] El-Ratel I.T., Attia K.A.H., El-Raghi A.A., Fouda S.F. (2020). Relief of the negative effects of heat stress on semen quality, reproductive efficiency and oxidative capacity of rabbit bucks using different natural antioxidants. Anim. Biosci..

[3] Plotuna A.M., Hotea I., Tîrziu E. (2025). Improving the Nutritional Properties of Rabbit Meat Through Dietary Supplementation with Linseed Meal, Fodder Yeast, and Selenium Yeast. Appl. Sci..

[4] Wongnaa C.A., Afful-Kwadam K., Asempah M.K., Hagan M.A.S., Awunyo-Vitor D. (2023). Is it profitable and viable to invest in commercialization of rabbit production? Implication on rural enterprise development. Sustain. Technol. Entrep. STAE.

[5] Zeferino C.P., Komiyama C.M., Fernandes S., Sartori J.R., Teixeira P.S.S., Moura A.S.A.M.T. (2013). Carcass and meat quality traits of rabbits under heat stress. Animal.

[6] Di Girolamo N., Toth G., Selleri P. (2016). Prognostic value of rectal temperature at hospital admission in client-owned rabbits. J. Am. Vet. Med. Assoc..

[7] Saxmose Nielsen S., Alvarez J., Bicout D.J., Calistri P., Depner K., Drewe J.A., Garin-Bastuji B., Gonzales Rojas J.L., Gortázar Schmidt C., EFSA Panel on Animal Health and Welfare (AHAW) (2020). Stunning methods and slaughter of rabbits for human consumption. EFSA J..

[8] Liang Z.L., Chen F., Park S., Balasubramanian B., Liu W.C. (2022). Impacts of heat stress on rabbit immune function, endocrine, blood biochemical changes, antioxidant capacity and production performance, and the potential mitigation strategies of nutritional intervention. Front. Vet. Sci..

[9] Ebeid T.A., Aljabeili H.S., Al-Homidan I.H., Volek Z., Barakat H. (2023). Ramifications of heat stress on rabbit production and role of nutraceuticals in alleviating its negative impacts: An updated review. Antioxidants.

[10] Abdel-Moneim A.M.E., Shehata A.M., Khidr R.E., Paswan V.K., Ibrahim N.S., El-Ghoul A.A., Aldhumri S.A., Gabr S.A., Mesalam N.M., Elbaz A.M. (2021). Nutritional manipulation to combat heat stress in poultry—A comprehensive review. J. Therm. Biol..

[11] Oladimeji A.M., Johnson T.G., Metwally K., Farghly M., Mahrose K.M. (2022). Environmental heat stress in rabbits: Implications and ameliorations. Int. J. Biometeorol..

[12] Huang D., Cai J., Zhang C., Jin R., Bai S., Yao F., Ding H., Zhao B., Chen Y., Wu X. (2023). Semen quality and seminal plasma metabolites in male rabbits (*Oryctolagus cuniculus*) under heat stress. PeerJ.

[13] Elbaz A.M., Zaki E.F., Salama A.A., Badri F.B., Thabet H.A. (2023). Assessing different oil sources efficacy in reducing environmental heat-stress effects via improving performance, digestive enzymes, antioxidant status, and meat quality. Sci. Rep..

[14] Emam K.R.S., Ali S.A., Morsy A.S., Fouda W.A., Elbaz A.M. (2024). Role of *Nannochloropsis oculata* supplement in improving performance, antioxidant status, blood metabolites, and egg quality of laying hens under hot environmental conditions. Sci. Rep..

[15] Abdel-Moneim A.M.E., Ali S.A., Sallam M.G., Elbaz A.M., Mesalam N.M., Mohamed Z.S., Abdelhady A.Y., Yang B., Elsadek M.F. (2025). Effects of cold-pressed wheat germ oil and *Bacillus subtilis* on growth performance, digestibility, immune status, intestinal microbial enumeration, and gene expression of broilers under heat stress. Poult. Sci..

[16] Abd El-Aziz A., Noreldin A., Elbaz A., Mishra B., Buonaiuto G., El-Sabrout K. (2025). Nano-selenium as a key supplement in rabbit nutrition: Physiological and productive benefits-a review. Trop. Anim. Sci. J..

[17] Abu Hafsa S.H., Centoducati G., Hassan A.A., Maggiolino A., Elghandour M.M., Salem A.Z. (2024). Effects of dietary supplementations of vitamin C, organic selenium, betaine, and pomegranate peel on alleviating the effect of heat stress on growing rabbits. Animals.

[18] El-Ratel I.T., Mekawy A., Hassab S.H., Abdelnour S. (2025). Enhancing growing rabbit heat stress resilience through dietary supplementation with natural antioxidants. BMC Vet. Res..

[19] Abidin Z., Khatoon A. (2013). Heat stress in poultry and the beneficial effects of ascorbic acid (vitamin C) supplementation during periods of heat stress. Worlds Poult. Sci. J..

[20] Abdel-Wareth A.A., Metwally A.E. (2020). Productive and physiological response of male rabbits to dietary supplementation with thyme essential oil. Animals.

[21] Puvača N., Tufarelli V., Giannenas I. (2022). Essential oils in broiler chicken production, immunity and meat quality: Review of *Thymus vulgaris*, *Origanum vulgare*, and *Rosmarinus officinalis*. Agriculture.

[22] Safwat A.M., Hassan O.A., El-Hady A.M.A., Kholif A.E., Sallam S.M., El-Zaiat H.M. (2021). Dietary supplementation of growing rabbits with lemongrass (*Cymbopogon citrates*) extract: Effects on performance, nutrient digestibility, anti-oxidative status, immune response and carcase characteristics. Ital. J. Anim. Sci..

[23] Elbaz A.M., El-Hawy A.S., Salem F.M., Lotfy M.F., Ateya A., Alshehry G., Alghamdi Y.S., Abd El-Hack M.E., Elolimy A.A., Abdelhady A.Y. (2025). Dietary incorporation of melittin and clove essential oil enhances performance, egg quality, antioxidant status, gut microbiota, and MUC-2 gene expression in laying hens under heat stress conditions. Ital. J. Anim. Sci..

[24] Abd El-Hack M.E., El-Saadony M.T., Saad A.M., Salem H.M., Ashry N.M., Ghanima M.M.A., Shukry M., Swelum A.A., Taha A.E., El-Tahan A.M. (2022). Essential oils and their nanoemulsions as green alternatives to antibiotics in poultry nutrition: A comprehensive review. Poult. Sci..

[25] Abdelsalam M., Fathi M. (2023). Improving productivity in rabbits by using some natural feed additives under hot environmental conditions—A review. Anim. Biosci..

[26] Elbaz A.M., Ashmawy E.S., Salama A.A., Abdel-Moneim A.M.E., Badri F.B., Thabet H.A. (2022). Effects of garlic and lemon essential oils on performance, digestibility, plasma metabolite, and intestinal health in broilers under environmental heat stress. BMC Vet. Res..

[27] Elbaz A.M., Ateya A., Youssef S.A., Arafa A.S., Sallam M.G., Abd El-Aziz A., Al-Rasheed M., Babaker M.A., Othman D.O., Gad G.G. (2025). Efficacy of in ovo feeding with *Lactobacillus acidophilus* and oregano essential oil in improving growth performance, immunity, microbial enumeration, and gene expression of ostrich chicks. Livest. Sci..

[28] Kačániová M., Čmiková N., Vukovic N.L., Verešová A., Bianchi A., Garzoli S., Ben Saad R., Ben Hsouna A., Ban Z., Vukic M.D. (2024). *Citrus limon* essential oil: Chemical composition and Selected biological properties focusing on the antimicrobial (in vitro, in situ), Antibiofilm, Insecticidal activity and preservative effect against *Salmonella enterica* inoculated in carrot. Plants.

[29] El-Saadony M.T., Saad A.M., Korma S.A., Salem H.M., Abd El-Mageed T.A., Alkafaas S.S., Elsalahaty M.I., Elkafas S.S., Mosa W.F., Ahmed A.E. (2024). Garlic bioactive substances and their therapeutic applications for improving human health: A comprehensive review. Front. Immunol..

[30] NRC (1977). Nutrient Requirements of Rabbits.

[31] Elbaz A.M., Althagafi H., Samy A., Arafa A.S., Abdelhady A.Y., Elkanawaty A.M., Alwutayd K.M., Shousha S., Hereba A.M., Sheikh A.I.E. (2025). Nano-Encapsulated Cumin Oil and *Bacillus subtilis* Enhance Growth Performance, Immunity, Oxidative Stability, and Intestinal Integrity in Growing Rabbits Under High Ambient Temperature. Vet. Sci..

[32] AOAC (1990). Official Methods of Analysis. Association of Official Analytical Chemists. https://archive.org/details/gov.law.aoac.methods.1.1990.

[33] Ibrahim N., Sabic E., Abu-Taleb A., Abdel-Moneim A. (2020). Effect of dietary supplementation of full-fat canola seeds on productive performance, blood metabolites and antioxidant status of laying Japanese quails. Braz. J. Poult. Sci..

[34] Imbabi T.A., Habashy W.S., Abol-Fetouh G.M., Labib M.M., Osman A., Elkelish A., Qurtam A.A., Tantawi A.A., Ahmed-Farid O. (2023). Enhancing semen quality, brain neurotransmitters, and antioxidant status of rabbits under heat stress by acacia gum, vitamin C, and lycopene as dietary supplements: An in vitro and in silico study. Ital. J. Anim. Sci..

[35] Hosny N.S., Hashem N.M., Morsy A.S., Abo-Elezz Z.R. (2020). Effects of organic selenium on the physiological response, blood metabolites, redox status, semen quality, and fertility of rabbit bucks kept under natural heat stress conditions. Front. Vet. Sci..

[36] Abdel-Moneim A.M.E., Elbaz A.M., Khidr R.E.S., Badri F.B. (2020). Effect of in ovo inoculation of *Bifidobacterium* spp. on growth performance, thyroid activity, ileum histomorphometry, and microbial enumeration of broilers. Probiotics Antimicrob. Proteins.

[37] Elbaz A.M., Farrag B., Farag B.F., Abdel-Moneim A.M.E. (2026). Effects of supplementing with *Nigella sativa* meal and selenium nano-particles on growth performance, immunity, microbial count, oxidative stability, and intestinal integrity-related gene expression in heat-stressed growing rabbits. Vet. Res. Commun..

[38] Rashid Z., Mirani Z.A., Zehra S., Gilani S.M.H., Ashraf A., Azhar A., Al-Ghanim K.A., Al-Misned F., Al-Mulahim N., Mahboob S. (2020). Enhanced modulation of gut microbial dynamics affecting body weight in birds triggered by natural growth promoters administered in conventional feed. Saudi J. Biol. Sci..

[39] Zhang J., McWhorter A.R., Khan S., Willson N.L., Chousalkar K.K. (2025). Characterization of *Lactobacillus* spp. isolated from layer hens as probiotic candidates. BMC Vet. Res..

[40] Ko H., Goo D., Lee J., Gyawali I., Katha H.R., Lee K.Y., Kim W.K. (2025). Effect of coated organic acids on growth performance, *Clostridium perfringens* colonization, gut integrity and immune response in broilers challenged with subclinical necrotic enteritis. Poult. Sci..

[41] Livak K.J., Schmittgen T.D. (2001). Analysis of relative gene expression data using real-time quantitative PCR and the 2−ΔΔCT method. Methods.

[42] Hieu T.V., Guntoro B., Qui N.H., Quyen N.T.K., Hafiz F.A.A. (2022). The application of ascorbic acid as a therapeutic feed additive to boost immunity and antioxidant activity of poultry in heat stress environment. Vet. World.

[43] Jang I.S., Ko Y.H., Kang S.Y., Lee C.Y. (2007). Effect of a commercial essential oil on growth performance, digestive enzyme activity and intestinal microflora population in broiler chickens. Anim. Feed Sci. Technol..

[44] Cerisuelo A., Marín C., Sánchez-Vizcaino F., Gómez E.A., De La Fuente J.M., Durán R., Fernández C. (2014). The impact of a specific blend of essential oil components and sodium butyrate in feed on growth performance and Salmonella counts in experimentally challenged broilers. Poult. Sci..

[45] Attia Y., Al-Harthi M., El-Kelawy M. (2019). Utilisation of essential oils as a natural growth promoter for broiler chickens. Ital. J. Anim. Sci..

[46] Albokhadaim I.F., Althnaian T.A., El-Bahr S.M. (2019). Gene expression of heat shoc kproteins/factors (HSP60, HSP70, HSP90, HSF-1, HSF-3) and antioxidant enzyme activities in heat stressed broilers treated with vitamin C. Pol. J. Vet. Sci..

[47] Ding X., Yu Y., Su Z., Zhang K. (2017). Effects of essential oils on performance, egg quality, nutrient digestibility and yolk fatty acid profile in laying hens. Anim. Nutr..

[48] Attia Y.A., Al-Khalaifah H., Ibrahim M.S., Abd Al-Hamid A.E., Al-Harthi M.A., El-Naggar A. (2017). Blood hematological and biochemical constituents, antioxidant enzymes, immunity and lymphoid organs of broiler chicks supplemented with propolis, bee pollen and mannan oligosaccharides continuously or intermittently. Poult. Sci..

[49] Kokoris J.C., Ruzic Z., Kanacki Z., Stojanovic S., Paras S., Milosevic V. (2024). Effects of vitamin C and early-age thermal conditioning on pituitary adrenocorticotropic hormone cells in broilers chronically exposed to heat stress: An immunohistomorphometric and hormonal study. Vet. Res. Forum.

[50] Zeweil H.S., Mandour M.A., Mahmoud S.A.S., El-Gendy Y.M. (2009). Effect of ascorbic acid supplementation on dose of rabbits exposed to different ambient temperatures. J. Appl. Sci. Res..

[51] Nikjoo M., Farhangi M., Patimar R., Adineh H., Alizadeh M. (2023). The protective effect of vitamin C on growth, digestive enzymes, immune response, and gill histology in Caspian roach (*Rutilus rutilus caspicus*) under diazinon stress. Aquac. Rep..

[52] Gopi M., Karthik K., Manjunathachar H.V., Tamilmahan P., Kesavan M., Dashprakash M., Balaraju B.L., Purushothaman M.R. (2014). Essential oils as a feed additive in poultry nutrition. Adv. Anim. Vet. Sci..

[53] Jimoh O.A., Ayedun E.S., Oyelade W.A., Oloruntola O.D., Daramola O.T., Ayodele S.O., Omoniyi I.S. (2018). Protective effect of soursop (*Annona muricata* linn.) juice on oxidative stress in heat stressed rabbits. J. Anim. Sci. Technol..

[54] Khan R., Ali S., Mumtaz S., Andleeb S., Ulhaq M., Tahir H.M., Khan M.K.A., Khan M.A., Shakir H.A. (2019). Toxicological effects of toxic metals (cadmium and mercury) on blood and the thyroid gland and pharmacological intervention by vitamin C in rabbits. Environ. Sci. Pollut. Res..

[55] Sumanu V.O., Naidoo V., Oosthuizen M., Chamunorwa J.P. (2023). A technical report on the potential effects of heat stress on antioxidant enzymes activities, performance and small intestinal morphology in broiler chickens administered probiotic and ascorbic acid during the hot summer season. Animals.

[56] Anoh K.U., Barje P.P., Iyeghe-Erakpotobor G.T., Akpa G.N. (2017). Growth performance of heat stressed rabbits fed diets supplemented with synthetic and organic antioxidants. Niger. J. Anim. Prod..

[57] Daader A.H., Al Sagheer A.A., Gabr H.A., Abd El Moniem E.A. (2018). Alleviation of heat-stress-related physiological perturbations in growing rabbits using natural antioxidants. Span. J. Agric. Res..

[58] Abdel-Wareth A.A., Taha E.M., Südekum K.H., Lohakare J. (2018). Thyme oil inclusion levels in a rabbit ration: Evaluation of productive performance, carcass criteria and meat quality under hot environmental conditions. Anim. Nutr..

[59] Huang Y., Ebrahimi H., Berselli E., Foti M.C., Amorati R. (2025). Essential Oils as Antioxidants: Mechanistic Insights from Radical Scavenging to Redox Signaling. Antioxidants.

[60] Elazab M.A., Khalifah A.M., Elokil A.A., Elkomy A.E., Rabie M.M., Mansour A.T., Morshedy S.A. (2022). Effect of dietary rosemary and ginger essential oils on the growth performance, feed utilization, meat nutritive value, blood biochemicals, and redox status of growing NZW rabbits. Animals.

[61] Liu L., Fu C., Li F. (2019). Acetate affects the process of lipid metabolism in rabbit liver, skeletal muscle and adipose tissue. Animals.

[62] Yilmaz E., Gul M. (2024). Effects of essential oils on heat-stressed poultry: A review. J. Anim. Physiol. Anim. Nutr..

[63] Lee K.W., Everts H., Kappert H.J., Frehner M., Losa R., Beynen A.C. (2003). Effects of dietary essential oil components on growth performance, digestive enzymes and lipid metabolism in female broiler chickens. Br. Poult. Sci..

[64] Abdel-Latif M., Sakran T., Badawi Y.K., Abdel-Hady D.S. (2018). Influence of Moringa oleifera extract, vitamin C, and sodium bicarbonate on heat stress-induced HSP70 expression and cellular immune response in rabbits. Cell Stress Chaperones.

[65] Alqhtani H.A., Elbaz A.M., Hegazy S.A., Abdelhady A.Y., Safhi F.A., Marzok M., Rizk M.A., Al-Rasheed M., Mohamed M.H., Abdel-Raheem S.M. (2026). Dietary Combined Thyme Meal and *Bacillus subtilis* to Promote Growth Performance, Immune Function, Gene Expression, Antioxidant Defense, and Cecal Microbiota in Growing Rabbits Under Heat Stress Conditions. Vet. Sci..

[66] Abdel-Hamid T.M., El-Tarabany M.S. (2019). Effect of bee pollen on growth performance, carcass traits, blood parameters, and the levels of metabolic hormones in New Zealand White and Rex rabbits. Trop. Anim. Health Prod..

[67] Patani A., Balram D., Yadav V.K., Lian K.Y., Patel A., Sahoo D.K. (2023). Harnessing the power of nutritional antioxidants against adrenal hormone imbalance-associated oxidative stress. Front. Endocrinol..

[68] Hajati H., Hassanabadi A., Golian A., Nassiri-Moghaddam H., Nassiri M.R. (2015). The effect of grape seed extract and vitamin C feed supplementation on some blood parameters and HSP70 gene expression of broiler chickens suffering from chronic heat stress. Ital. J. Anim. Sci..

[69] Macvanin M.T., Gluvic Z., Zafirovic S., Gao X., Essack M., Isenovic E.R. (2023). The protective role of nutritional antioxidants against oxidative stress in thyroid disorders. Front. Endocrinol..

[70] Hirakawa R., Nurjanah S., Furukawa K., Murai A., Kikusato M., Nochi T., Toyomizu M. (2020). Heat stress causes immune abnormalities via massive damage to effect proliferation and differentiation of lymphocytes in broiler chickens. Front. Vet. Sci..

[71] Hu H., Bai X., Shah A.A., Wen A.Y., Hua J.L., Che C.Y., He S.J., Jiang J.P., Cai Z.H., Dai S.F. (2016). Dietary supplementation with glutamine and γ-aminobutyric acid improves growth performance and serum parameters in 22- to 35-day-old broilers exposed to hot environment. J. Anim. Physiol. Anim. Nutr..

[72] Wu Q.J., Liu N., Wu X.H., Wang G.Y., Lin L. (2018). Glutamine alleviates heat stress-induced impairment of intestinal morphology, intestinal inflammatory response, and barrier integrity in broilers. Poult. Sci..

[73] Carr A.C., Maggini S. (2017). Vitamin C and immune function. Nutrients.

[74] Khan M., Mushtaq M., Usman M., Rahman M.A.U., Quan G. (2025). Oxidative stress-induced cytotoxicity and the role of dietary antioxidants in farm animals: A review. Adv. Redox Res..

[75] Gęgotek A., Skrzydlewska E. (2022). Antioxidative and anti-inflammatory activity of ascorbic acid. Antioxidants.

[76] Elbaz A.M., Al-Rasheed M., El-Naggar K., Tahoun A., Arafa A.S., Kandeel M., El-Sharkawy H. (2025). Effect of Supplementation with Bee Antibacterial Peptides and Turmeric Essential Oil on Microbial Enumeration and Intestinal Integrity-Related Genes, Immunity, and Performance in Heat-Stressed Broilers. Avian Dis..

[77] Attia Y.A., El-Naggar A.S., Abou-Shehema B.M., Abdella A.A. (2019). Effect of supplementation with trimethylglycine (betaine) and/or vitamins on semen quality, fertility, antioxidant status, DNA repair and welfare of roosters exposed to chronic heat stress. Animals.

[78] Güven L., Behbudbayli U., Ertürk A., Hancı H., Yılmaz B., Kaya Y., Gülçin İ. (2023). Determination of antioxidant, antimicrobial, anticholinesterase, antityrosinase, antidiabetic and antiglaucoma activities of essential oils from three different *Thymus* species and their chemical characterization by GC-MS analysis. J. Essent. Oil Bear. Plants.

[79] Raspa M., Paoletti R., Scavizzi F. (2025). Ascorbic acid 2-glucoside improves survival, quality, and fertility of frozen-thawed C57Bl/6J and C57Bl/6N mouse spermatozoa. J. Androl..

[80] Atsain-Allangba M.R. (2026). Antioxidant profile and antimicrobial activity of ascorbic acid: An in vitro study. Sci. Struct. Matière.

[81] Zhao G., Li P., Mu H., Li N., Peng Y. (2021). L-ascorbic acid shapes bovine *Pasteurella multocida* serogroup A infection. Front. Vet. Sci..

[82] Mumtaz S., Ali S., Tahir H.M., Kazmi S.A.R., Mughal T.A., Younas M. (2023). Evaluation of antibacterial activity of vitamin C against human bacterial pathogens. Braz. J. Biol..

[83] Pugliese G., Losacco C., Laudadio V., Schiavitto M., Tufarelli V. (2024). Plant extracts and essential oils in rabbit diet: A practical green-way for sustainable and resilient production systems. J. Hell. Vet. Med. Soc..

[84] Hassan M.A., Abo-Elmaaty A.M., Zaglool A.W., Mohamed S.A., Abou-Zeid S.M., Farag M.R., Alagawany M., Di Cerbo A., Azzam M.M., Alhotan R. (2023). Origanum vulgare essential oil modulates the AFB1-induced oxidative damages, nephropathy, and altered inflammatory responses in growing rabbits. Toxins.

[85] Jimoh O.A., Ayodele A.D., Ojo O.A., Okin-Aminu H.O., Olarotimi O.J. (2024). Effects of turmeric, ginger, cinnamon, and garlic essential oils on HSP70, NFκB, oxidative DNA damage, inflammatory cytokines, and oxidative markers in broiler chickens. Transl. Anim. Sci..

[86] Madkour M., Hosny M., Aboelazab O., Fahmy S., Rayan G., Ali A.H., Abdel Moati Y.A., Elolimy A.A., Fathi M. (2025). Dietary encapsulated essential oils improve growth performance and regulate stress-responsive genes in heat-stressed broilers. J. Anim. Sci..

[87] Ruan D., Fan Q., Fouad A.M., Sun Y., Huang S., Wu A., Lin C., Kuang Z., Zhang C., Jiang S. (2021). Effects of dietary oregano essential oil supplementation on growth performance, intestinal antioxidative capacity, immunity, and intestinal microbiota in yellow-feathered chickens. J. Anim. Sci..

[88] Elbaz A.M., El-Sonousy N.K., Arafa A.S., Sallam M.G., Ateya A., Abdelhady A.Y. (2024). Oregano essential oil and *Bacillus subtilis* role in enhancing broiler’s growth, stress indicators, intestinal integrity, and gene expression under high stocking density. Sci. Rep..

